# The Reserva de la Biosfera Barranca de Metztitlán (Hidalgo): An illustrated checklist of bromeliads and orchids and their high levels of Mexican endemisms

**DOI:** 10.3897/phytokeys.118.31603

**Published:** 2019-03-12

**Authors:** Claudia T. Hornung-Leoni, Yesenia J. Chavarria-Olmedo, Ivón M. Ramírez-Morillo

**Affiliations:** 1 Centro de Investigaciones Biológicas, Instituto de Ciencias Básicas e Ingeniería, Herbario HGOM. Universidad Autónoma del Estado de Hidalgo. Pachuca, Hidalgo. Mexico Universidad Autónoma del Estado de Hidalgo Mineral de La Reforma Mexico; 2 Herbario CICY, Centro de Investigación Científica de Yucatán, A.C. Calle 43 # 130 x 32 y 34, Colonia Chuburná de Hidalgo 97205, Mérida, Yucatán, Mexico Centro de Investigación Científica de Yucatán Mérida Mexico

**Keywords:** Metztitlán, Bromeliaceae, Orchidaceae, flora, endemism, conservation, rare species

## Abstract

This study presents a list of species of the two most important families with epiphytic elements, Bromeliaceae and Orchidaceae, from the Reserva de la Biosfera Barranca de Metztitlán (RBBM), the largest Reserve in Hidalgo, Mexico. Thirty-four species are included, 26 corresponding to species in three genera of bromeliads, and eight species in six genera of orchids. The new records represent 26.5% of the total listed in the area; nine of them are new records for the Reserve (RBBM) and one is new for Hidalgo State. This study reveals that endemism for both families is very important in the Reserve (55.88%), since it includes 13 Mexican bromeliads, of which two are endemic to Hidalgo and one to the Reserve, and three orchids, two endemic to Mexico and one to the Reserve. We found species with different types of relative abundance: rare (16) and occasional (7). Additionally, we include information about the category (IUCN, CITES, NOM-059-SEMARNAT) as well as uses reported in the literature for the species in the RBBM. The checklist is strictly based on information obtained from deposited herbarium specimens as well as from those collected during fieldwork. We suggest that a conservation plan (*in situ* and *ex situ*) for the RBBM is important and necessary. The predominant habit for both families is epiphytic (17 species); even though there are terrestrial (7) and saxicolous (2), and the remaining are facultative species (8). Nine species are included in some risk category. The present work is the most complete and updated list of Bromeliaceae and Orchidaceae for this important natural area in the Mexican State of Hidalgo. However, more fieldwork is needed to document the biodiversity of the area in general and its flora in particular, as a way to highlight the importance of protected areas in preserving biodiversity.

## Introduction

**Figure 1.**
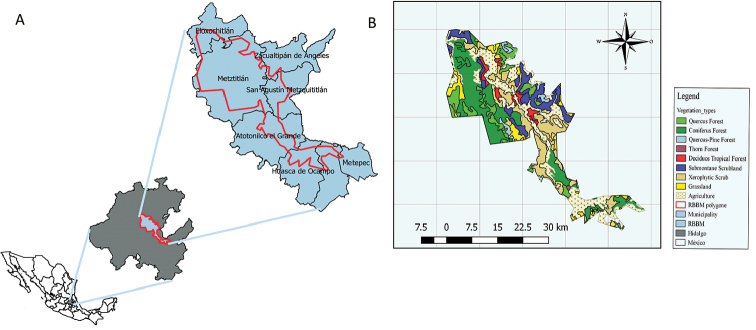
Map of the study area indicating: **A** Location of the RBBM in Hidalgo State and municipalities, and **B** Vegetation types.

The Reserva de la Biosfera Barranca de Metztitlán (from here on RBBM) is located in the east-central region of the Mexican State of Hidalgo and is considered to be the most important protected natural area in Hidalgo State (see Figure 1). RBBM is considered a Pleistocene refuge of the Mexican desert biota since it has characteristics that show the strong relation that existed in the past with the Chihuahua and Sonora deserts, and is currently functioning as a biological corridor of arid zones in the central highlands of the country (CONANP 2003).

Tolantongo is the only area in Hidalgo State that has a formal floristic inventory (Hiriart and González-Medrano 1983), while RBBM flora is not well known. The management plan for RBBM (CONANP 2003) includes 11 bromeliads and six species of orchids. However, a careful study of this list shows some misidentifications. Moreover, the information used to produce the list was mainly taken from literature, which makes it impossible for specialists to review the species identification since the cited taxa are not supported by specimens housed in herbaria.

A preliminary orchid list of Hidalgo (Chavarria et al. in prep.) positioned this state in an important place (around the 5^th^ national position) for orchid species richness in Mexico. However, the RBBM has been very poorly explored and, in our opinion, it is vital to carry out floristic studies that may enhance research in the State and improve management plan strategies. The present study aims to contribute to this goal, providing an updated list of bromeliads and orchids of RBBM.

Some previous publications on the Bromeliaceae of Hidalgo (Pintado 2010, Ceja-Romero et al. 2010, Hornung-Leoni and Pintado 2011, Hornung-Leoni 2017) have added information about the family in the region, but the present approach is focused on the RBBM. Recent studies in Mexican Bromeliads have documented 442 species for the country and 35 for Hidalgo State (Espejo-Serna and López-Ferrari 2018). On the other hand, Hornung-Leoni (2017) in the Hidalgo Bromeliad flora reports 47 species, ranking it in 8^th^ position among the richest states for bromeliads species in Mexico.

## Methods

### Area of study

The RBBM includes several biogeographical regions (“Sierra Madre Oriental” and “Eje Neovolcanico Transmexicano”) and presents at least six, mainly dry vegetation types: xerophytic scrub, submontane scrubland, deciduous tropical forest, coniferous forest, grassland and riparian vegetation (Rzedowski 1978, Morrone 2001, CONANP 2003). In order to elaborate the map (Figure 1), we also considered the information about the use of soil and nine types of vegetation (e.g. agricultural, *Quercus* forest, among others) provided by INEGI (2017).

The RBBM has an area of 960.42 km^2^ and is located in the eastern-central region of Hidalgo State (19°35'52"–21°25'00"N and 97°57'27"– 99°51'51"W) (Figure 1). The topography is rugged and the altitudinal gradient ranges from 1100 to 2600 m a.s.l., which determines the presence of different climates and vegetation types. The climate varies from semi-dry to temperate dry, with an annual average temperature of 18–22 °C and average annual rainfall of 400–700 mm (CONANP 2003). This area constitutes a biological corridor between the Nearctic and Neotropical regions; it is located in northern Mexico and includes eight municipalities.

### Fieldwork and herbaria

Fieldwork in bromeliads has been conducted in RBBM since 2008. All collected specimens are housed in the Centro de Investigaciones Biológicas Herbarium (HGOM) of the Universidad Autónoma del Estado de Hidalgo. On the other hand, orchid data from the herbarium HGOM or from fieldwork, started to be collected and data-based in 2010.

For species identifications we used specialized botanical literature: Espejo-Serna et al. (2005, 2010), Pintado (2010), Ceja-Romero et al. (2010) and Hornung-Leoni (2017) for bromeliads and we reviewed Hágsater et al. (2005) and Soto-Arenas et al. (2007) for orchids. Dr. Gerardo Salazar identified the orchid specimens in HGOM. Bromeliads were identified by C. Hornung-Leoni and I. Ramirez-Morillo.

All our reports are strictly based on herbarium specimens deposited in several herbaria (AMES, AMO, CHAP, CICY, ENCB, GH, F, IEB, IBUG, MICH, MEXU, MO, UAMIZ, US, SEL, WU). Additionally, we used high resolution images from the same herbaria as well as online databases to complement some data (e.g. distribution and altitudes) (Table 1).

For authors, we followed Brummitt and Powell (www.ipni.org); for synonymy, we used The Plant List (www.theplantlist.org) and for the species names, we consulted Tropicos (http://tropicos.org/).

Information about endemism was obtained from previous studies about recently discovered new species (Espejo-Serna et al. 2004, Espejo-Serna 2012, Ramírez et al. 2015, Villaseñor 2016); as for risk categories, we consulted IUCN (2017), NOM-059-SEMARNAT (SEMARNAT 2010) and CITES (2017) (www.cites.org). For species with restricted distribution inside the RBBM limits (like *Tillandsiatortilis*, *T.mauryana* and *Sotoaconfusa*), GeoCAT tool interface program (Bachman et al. 2011) was employed in order to calculate risk category *sensu* IUCN criteria, considering extent of occurrence (EOO) and area of occupancy (AOO) based on coordinates, for those cases in which we found at least three different collecting points as required by the software.

## Results

**Figure 2.**
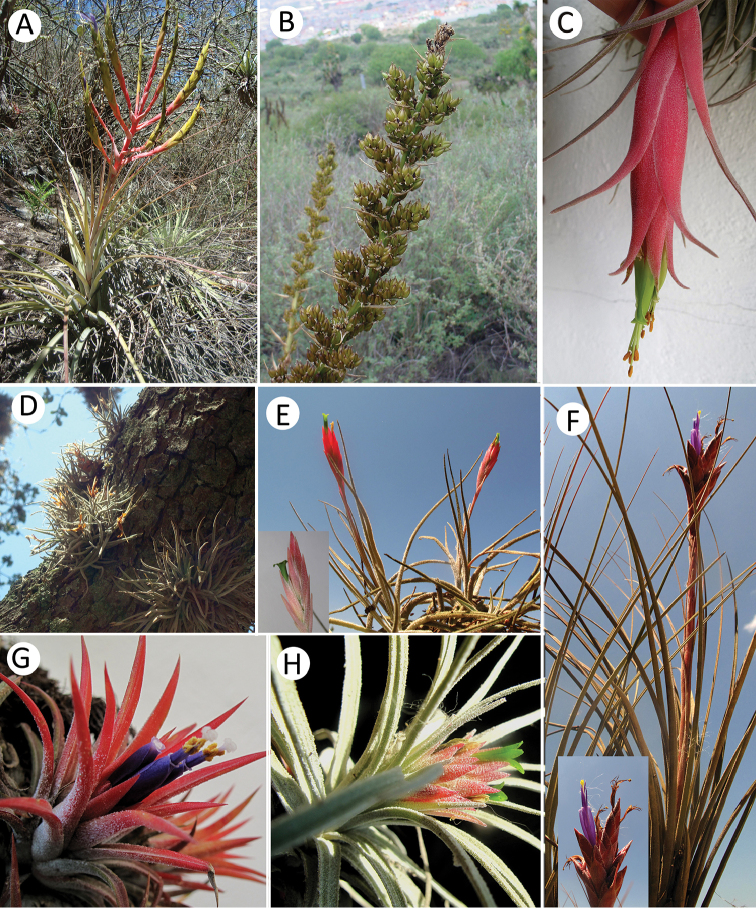
Species of bromeliads in Reserva de la Biosfera Barranca de Metztitlán (RBBM): **A***Tillandsiainopinata***B***Hechtiapodantha***C***Tillandsiaerubescens***D***Tillandsiarecurvata***E***Tillandsiatortilis***F***Tillandsiajuncea***G***Tillandsiaionantha***H***T.mauryana*. Photographs: **A–H** by Hornung-Leoni.

**Figure 3.**
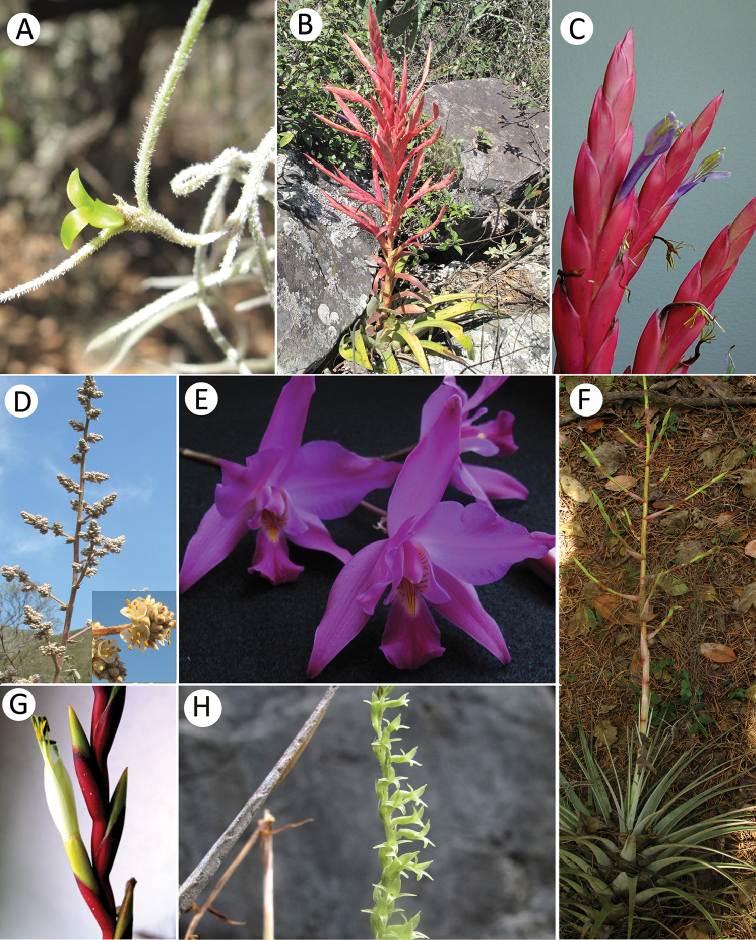
Species of Bromeliaceae (*cont*.) (**A–D, F, G**) and Orchidaceae (**E, H**) in Reserva de la Biosfera Barranca de Metztitlán (RBBM): Bromeliaceae: **A***Tillandsiausneoides***B***Tillandsiaparryi***C***Tillandsiadeppeana***D***Hechtiaglomerata***E***Laeliagouldiana***F***Tillandsiapringlei***G***Tillandsiaalbida***H***Mesadenuspolyanthus*. Photographs: **A–D** and **F–H** by Hornung-Leoni. Photograph E by Chavarria-Olmedo.

**Figure 4.**
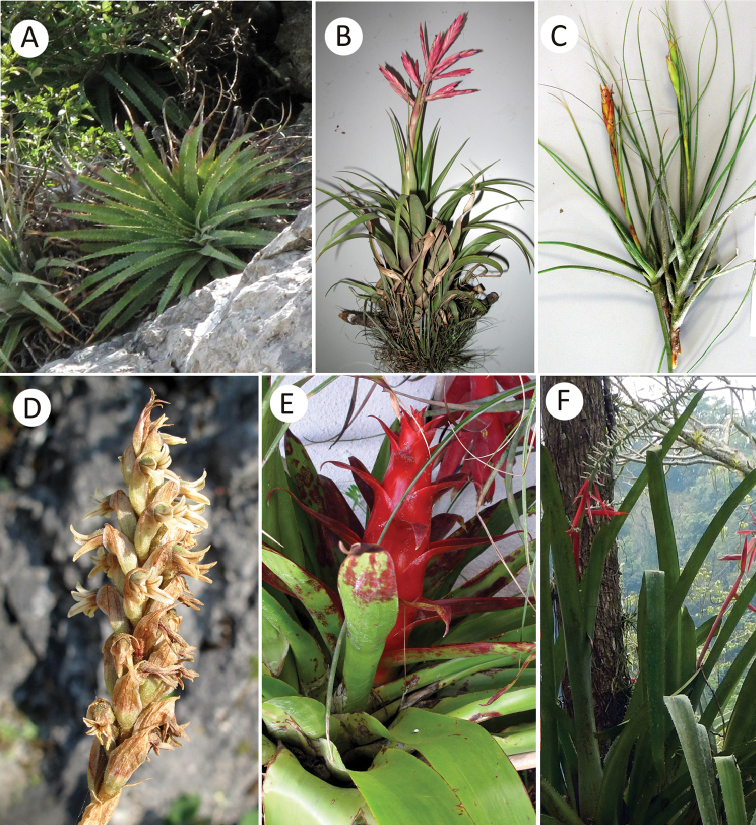
Bromeliaceae and Orchidaceae (*cont*.) in Reserva de la Biosfera Barranca de Metztitlán (RBBM) (*cont*.) **A***Hechtiadeceptrix***B***Tillandsiagymnobotrya***C***Tillandsiaschiedeana***D***Aulosepalumpyramidale***E***Tillandsiaimperialis***F***Aechmeabracteata*. Photographs: **A–E** by Hornung-Leoni and **F** by I. Ramírez-Morillo.

A total of 34 species from both families (Table 1, Figures 2–4) were found in RBBM. Of these, 26 species belong to Bromeliaceae classified into three genera and eight are orchid species distributed among six genera (Table 1). Three orchids and six bromeliads were added as new records of RBBM. The most representative genus in bromeliads was *Tillandsia* with 21 species, followed by *Hechtia* with five species. *Laelia* is the species-richest genus of orchids.

Namely, from the xerophytic area we are reporting orchids like *Laelia* and *Cyrtopodium* species and bromeliads like *Hechtia* spp., *Tillandsiaalbida* and *T.inopinata*. In humid environments, orchids like *Aulosepalum*, *Sarcoglottis* and *Malaxis*, and bromeliads like *T.violacea*, *T.imperialis* and *T.deppeana*, are found.

**Figure 5.**
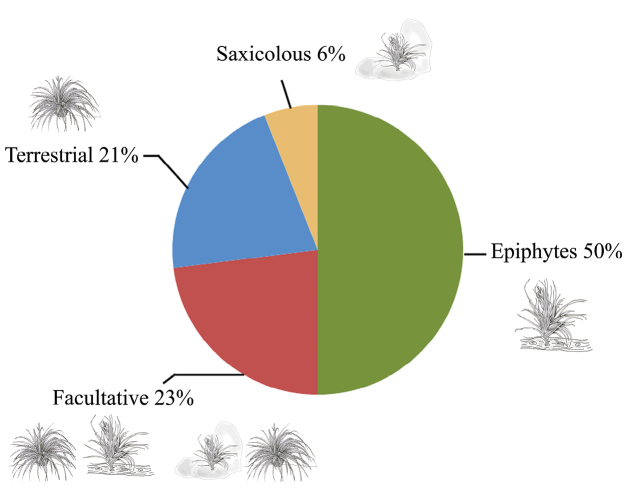
Habits in species present in RBBM

Epiphytic habit is predominant in both families corresponding to 50% of the species (see Figure 5); however, terrestrial and facultative epiphytic species are also important in the RBBM. Hechtias grow both as saxicolous and terrestrials. *Aechmeabracteata* was found only as an epiphyte, while *Tillandsiamauryana* prefers the saxicolous habit. As facultative we listed individuals that can be found in two different categories (e.g. saxicolous and epiphytes or saxicolous and terrestrial).

Different levels of endemism are present in the RBBM including both families (Table 1), totaling 55.88% of the recorded species. Thirteen of the bromeliads found in the Reserve are endemic to Mexico (like *Tillandsiatortilis*, *Hechtialepidophylla*); two are endemic to Hidalgo (*H.deceptrix* and *T.mauryana*) and one bromeliad is endemic to the Reserve (*Hechtia* sp.). For orchids, a total of three species present some level of endemism: two are endemic to Mexico (*Sotoaconfusa* and *L.speciosa*), and one (*Laeliagouldiana*) is present only inside the RBBM even though it has been considered *extinct in situ* in the NOM-059-SEMARNAT (SEMARNAT 2010). This species is only found on “Mesquite” trees (*Prosopislaevigata*, Fabaceae: Mimosoideae) which grow near local people’s houses inside the RBBM (Soto-Arenas et al. 2002).

The categories of threatened taxa (IUCN (2017), CITES (2017), NOM-059-SEMARNAT (SEMARNAT 2010) found in literature are reported in Table 1.

**Table 1.** List of the bromeliad and orchid species recognized in this study at the RBBM. Taxa are arranged alphabetically in families and genera; the collector’s name, number and herbaria are indicated. For more details of vouchers and distribution of species, see Suppl. material 1. Species marked with * indicate a new register of RBBM. Endemism: MEX: endemic to Mexico, HGO: endemic to Hidalgo State, and RBBM: endemic to the Reserve. NOM-059-SEMARNAT Mexican categories: threatened (A), probably extinct in the wild (E), in danger of extinction (P), and subject to special protection (Pr). Habit: E= epiphytic, T=terrestrial, S= saxicolous. See Suppl. material 1 for more details.

Species	Vouchers	Examined Herbariums	Endemism	Habit	Relative species abundance	Threatened taxa
** Bromeliaceae **						
1. *Aechmeabracteata* (Sw.) Griseb.	AGPP 114	HGOM		E	rare	
2. *Hechtiadeceptrix* I. Ramírez & C.T. Hornung *	CTHL 1598	HGOM	HGO	T	rare	VU (IUCN)
3. *H.glomerata* Zucc.	AGPP 178, CTHL 1249, CTHL 1329, CTHL 1330, CTHL 1430, CTHL 1431, CTHL 1432, CTHL 1433, CTHL 1564	HGOM		T/S	abundant	
4. *H.lepidophylla* I. Ramirez	LGQ 1189	IEB	MEX	T	rare	
5. *H.podantha* Mez	G 5291, UG 2135,	MEXU	MEX	T	abundant	
6. *Hechtia* sp. *	AGPP 118	HGOM	RBBM	T	rare	
7. *Tillandsiaalbida* Mez & Purpus	AGPP 66, AGPP 122, AGPP 173, ARLF 2132, AES 1782, EM 38486, CTHL 1153, CTHL 1331, CTHL 1435, CTHL 1603,	HGOM, MEXU	MEX	T/S	common	
8. *T.atroviridipetala* Matuda	ARLF 3279, JR 19504	UAMIZ, MEXU	MEX	E	rare	
9. *T.bartramii* Elliott	AGPP 68, CTHL 1559, CTHL 1605	HGOM		E	occasional	
10. *T.deppeana* Steud.	AGPP 121, JLLG 412, CS 53,	HGOM, CHAP, UAMIZ	MEX	E	frequent	
11. *T.erubescens* Schltdl. *	CTHL 1572	HGOM	MEX	E	occasional	
12. *T.festucoides* Brongn. ex Mez *	AGPP 124	HGOM		E	rare	Pr category NOM-059
13. *T.gymnobotrya* Baker	AGPP 119, JC 1954, JC 1960, JLLG 494, JLLG 410, JC 1296, JC 1301, CS 59	UAMIZ, MEXU, IEB, CHAP	MEX	E	rare	
14. *T.imperialis* É. Morren ex Mez	AGPP 120, JC 1969, AES 6661, JC 1960	HGOM, UAMIZ		E	rare	A category NOM-059
15. *T.inopinata* Espejo, López-Ferr. & W. Till	AGPP 63, AGPP 64, AGPP 112, AGPP 113, AGPP 115, AGPP 136, AGPP 141, CTHL 1187, CTHL 1240, 1241, CTHL 1242, CTHL 1541, CTHL 1542, CTHL 1555, ARLF 3275, LGQ 1202, LGQ 2567, ALB 37, PM 5765. SS 76/30, SS 77/7, HEM 2471, JR 19505	HGOM, ENCB, MEXU, GH, WU, US, UAMIZ	MEX	T/S/E	abundant	
16. *T.ionantha* Planch.	AGPP 130, AGPP 134, AGPP 138, CTHL 1505, CTHL, 1506, CTHL 1508, CTHL 1522, CG 22	HGOM, ENCB		E/S	frequent	LC IUCN
17. *T.juncea* (Ruiz & Pav.) Poir.	AGPP 110, AGPP 111, AGPP 142, AGPP 174, JC 1966, EG 5131, EG 5276, BL 2645. ARLF 3274, JC 1292, TBC 65815, FG 8402, LG 1203, ARLF 3281, MM 2533, HEM 2469, JR 19503, CTHL 1540, CTHL 1543, CTHL 1544, CTHL1547, CTHL 1604	HGOM, UAMIZ, CHAP, MEXU, ENCB, US		E	abundant	
18. *T.lepidosepala* L.B.Sm.	HEM 4221, ARLF 2131	MICH, UAMIZ	MEX	E	rare	
19. *T.mauryana* L.B.Sm.	CTHL 1328; CTHL 1557, CTHL 1561, CTHL 1570, CTHL 1666, AGPP 140, YJCO 75; DG 2, ARLF 2133, PM 5747, JC 1768, JC1967, DG 2, ALS *sn*, ARLF 2133, PM 5747, EZ *sn.*	HGOM, MEXU,SEL UAMIZ, F, GH, WU	HGO	S	occasional	CITES, IUCN: EN (EOO/AOO)
20. *T.parryi* Baker	JV 1961, RE 942403, JC 1298, JC 1300, ARLF 3282, JLLG 411	UAMIZ, M, CICY, IBUG, MEXU	MEX	E	rare	
21. *T.pringlei* S.Watson *	CTHL 1434, CTHL 1560	HGOM	MEX	E	rare	
22. *T.recurvata* (L.) L.	AGPP 67, AGPP 117, AGPP 125, AGPP 177, AGPP 179, LCR 426, EGN 5122, EGN 5222, EGN 5262, ARLF 3277, JMQ*sn*, sc*sn*, ARLF 3353, RR*sn*, ID 757, MF 167, CTHL 1507	MEXU, CHAP, UAMIZ		E	abundant	
23. *T.schiedeana* Steud. *	AGPP 132, AGPP 175	HGOM		E	abundant	
24. *T.tortilis* Klotzsch ex Baker *	CTHL 1600, CTHL 1601	HGOM	MEX	E	rare	IUCN: EN (AOO)/ NT (EOO)
25. *T.usneoides* (L.) L.	AGPP 116, AGPP 143,JJC 1956, EG 5262, FGM 7963, FGM 10359, ARLF 3280, ARLF 3283, CTHL 1545	UAMIZ, XAL, MEXU, UAMIZ		E/S	abundant	
26. *T.violacea* Baker	JLLG 507	MEXU	MEX	E	occasional	
** Orchidaceae **						
1. *Aulosepalumpyramidale* (Lindl.)M.A.Dix & M.W.Dix *	AMR 1513, CTHL 1333; CTHL 1334, CTHL 1339	UAMIZ, HGOM		T	rare	
2. *Cyrtopodiummacrobulbon* (La Llave & Lex.) G.A.Romero & Carnevali	AES 2431, AES 2432, AES 243; JAM 6	UAMIZ, HGOM		S	occasional	CITES
3. *Laeliaanceps* Lindl.	FGM 8438, JAM 15	MEXU, HGOM		E/ S	occasional	P category NOM-059 CITES
4. *L.gouldiana* Rchb.f.	AES 2213, EH 6000, WBT 2500, WBT 6085, GAS 8194	UAMIZ, US, ENCB, AMO, MEXU	RBBM	E	rare	E category NOM-059 CITES
5. *L.speciosa* (Kunth) Schltr.	RHM 6078, GAS 8194, ARLF 3273	MEXU, UAMIZ, MO	MEX	E/ S	occasional	Pr category NOM-059 CITES
6. *Mesadenuspolyanthus* (Rchb.f.) Schltr. *	CTHL 1552, CTHL 1562	HGOM		T/ S	rare	
7. *Sarcoglottisschaffneri* (Rchb.f.) Ames *	CTHL 1255, CTHL 1708	HGOM		T	occasional	
8. *Sotoaconfusa* (Garay) Salazar	JG 2194	AMES	MEX	T	rare	CITES, IUCN: LC (EOO) / EN (AOO)

Collector’s legend: AGPP: A.G. Pintado Peña; ALB: A. López B.; AMR: A. Mendoza R.; ARLF: A.R. López-Ferrari; CG: C. García; CS: C. Sanchez; JCR: J. Ceja R.; CTHL: C.T. Hornung-Leoni; EG: E. Guízar; EH: E. Hágsater; EM: E. Matuda; FGM: F. González Medrano; FM: F. Miranda; G: Guízar; GAS: G.A. Salazar; HEM: H. E. Moore jr.; ID: I. Díaz; JAM: J. Ángeles Mota; JG: J. González; JLLG: J. L. López G.; JMQ: J. M. Quintanilla; JR: J. Rzedowski; LB: B. Leuenberger; LGQ: L. González Q.; MASA: M.A. Soto Arenas; MF: M. Flores; MM: M. Medina; PG: P. Gold; PM: P. Maury; RE: R. Ehlers; sc: without collector; RHM: R. Hernández Magaña; RR: R. Robledo; SS: S. Schatzl; TBC: T.B. Croat; UG: U. Guzmán Cruz; YJCO: Y.J. Chavarria Olmedo; WBT: W.B. Thurston.

## Discussion

After revising the available literature for Hidalgo, we found that seven species of bromeliads were mentioned for the RBBM (Espejo-Serna et al. 2004) and 12 species in the management plan (CONANP 2003); eight species were cited by Ceja-Romero et al. (2010) and 24 bromeliads by Pintado (2010). However, it is important to point out that the main goal of these studies is not the bromeliad flora of the RBBM and that, in some cases, it was impossible to establish the existence of some species inside the limits of the RBBM. On the other hand, the taxonomic status of the species included in the management plan (CONANP 2003) needs to be revised, since some of the cited names are synonymous or misplaced species (see details below).

For orchids, although the management plan CONANP (2003) reported six species, Soto-Arenas and Solano-Gómez (2007) as well as Ceja-Romero et al. (2010), cited only three species of Laelias (*Laeliaautumnalis*, *L.gouldiana*, *L.speciosa*).

In neotropical studies, both families here included are very representative as far as richness is concerned (Rodrigues et al. 2017). Therefore, new studies are required to improve the characterization of the biodiversity of the region and to promote the species protection inside the RBBM.

### Species in RBBM and Endemism

It is important to emphasize that several of the species found by us at RBBM were mentioned before in the literature (CONANP 2003, Vincenzo et al. 2012), but their presence based on previous reports is impossible to be confirmed since many of them lack herbarium specimens. Therefore, ours constitutes the first confirmed and verifiable report, since it is supported by specimens housed in public collections.

In the management plan of the RBBM (CONANP 2003), 12 bromeliads and orchids are cited; however, some nomenclatural problems need to be mentioned. First, among bromeliads they reported *Tradescantia* sp., a member of Commelinaceae, which needs to be discarded. Secondly, some of the cited species’ names are currently treated as synonymous or are invalidly published (*sensu* Plant List and Tropicos): for example, *Tillandsiaehrenbergiana* Hemsl., is a synonym of *T.utriculata* but we have no records of this last species in the study area. *Tillandsiaehrenbergiana* Klotzsch ex Baker is synonymous with *T.tortilis* and for this taxon we have several records. Another case is that of *Tillandsiabenthamiana* Klotzsch ex Baker, currently treated as a synonym of *T.erubescens*, a taxon here reported for the RBBM. Tillandsiabenthamianavar.andrieuxii Mez is recognized as *T.andrieuxii*, a species for which we also did not find specimens deposited in herbaria because we did not collect it in the study area. For these reasons, we consider that the correct names for the species previously cited for the area (CONANP 2003) are *T.tortilis* and *T.erubescens*. Moreover, even if they are synonyms, these species have not been found in herbaria until now. So, *T.tortilis* and *T.erubescens* were considered as new records because our vouchers are the first ones collected for both species.

For orchids, in the management plan (CONANP 2003) species like *Cyrtopodiummacrobulbon*, *C.punctatum* and *Epidendrumramosissimum* Ames & C. Schweinf. were reported, even though the last species is only documented for Costa Rica; it is probably a case of misidentification and will not be clarified since there is no specimen or image to verify this report. Other orchid species reported for the RBMM (*sensu*Vincenzo et al. 2012), are *Anathallisminutalis*, *Epidendrumramosum*, *Laeliaautumnalis*, *L.gouldiana*, *L.anceps*, and *L.speciosa*. The management plan reports the presence of *E.ramosissimum* that Vincenzo et al. (2012) recognize as *E.ramosum*, but both are only literature references. For this reason they are not included in the present study. For *Pleurothallisminutalis* (*sensu*CONANP 2003 and Vincenzo et al. 2012), currently treated as *Anathallisminutalis*, its presence has not been collected yet for the orchid flora of Hidalgo State (Chavarria et al. in prep.).

Since RBBM provides many habitats, vegetation types and different substrates, the Reserve is a relevant area for the preservation of the flora in the state and as more collections become available the numbers reported here for endemism may change, since they are affected by the collection effort as well as the growing knowledge about species distribution. The number of species present in the RBBM is considerable, in relation to the area occupied by this reserve within the State. Nevertheless, this fact was not unexpected because it is the largest Reserve of Hidalgo State and includes several vegetation types as well as a variety of climatic and environmental conditions. In spite of this richness, in our opinion the diversity of bromeliads and mainly orchids has been underestimated. This is unsurprising considering that Rzedowski (2015) mentioned that knowledge of Mexican flora is still a work in progress with approximately 30% of species still unknown.

The greatest richness in the RBBM is represented by *Laelia* for orchids and *Tillandsia* for bromeliads, the latter being the most diverse genus of the bromeliads in Hidalgo (Hornung-Leoni 2017). Some of them are still poorly collected. For example, *Tillandsiatortilis*, has been reported before outside the limits of Metztitlán (Ceja-Romero et al. 2010), but we found this species inside the RBBM near its borders. We consulted some local people who commented that they do not have any interest in this plant since they only consider it as a parasite and, in some cases, it is removed from trees. Another species, *T.recurvata*, is very abundant in *Prosopis* spp. trees and other bushes in the Reserve. Sterile individuals of *Hechtiadeceptrix*, a recently described species endemic to Hidalgo (Ramírez et al. 2015), were observed inside the RBBM (on the road from Metztitlán to Tolantongo). This finding extends the distribution of the species reported earlier by Ramírez et al. (2015) for the Carso Huasteco within the Sierra Madre Oriental Province (*sensu*Morrone 2014). However, the presence of this species inside the RBBM was expected since it is located in the same biogeographic area. Other species that we expected to find are *Pitcairniaringens* (Bromeliaceae) and *Dicromanthuscinnabarinus* (Orchidaceae), since both of them have been reported near the limits of the RBBM, despite not being vouchered up to now.

The RBBM can be cited as an example of how a Reserve created for other purposes (e.g. to preserve Cactaceae), can be a refuge for other endemic, rare, and even new species like bromeliads and orchids. After completing floristic inventories, conservational strategies should be developed, counting on people who live inside the Reserve to assist in the species/habitats conservation. In fact, people living inside the reserve are very conscious of the natural resources they have, and may take part to protect the biota. Additional strategies may include ex-situ cultivation of threatened species and the promotion of campaigns to avoid extraction of individuals from their habitat.

### Habit predominance

Even though epiphytic species are dominant in RBBM, some species like *T.juncea* can be facultative (epiphytes and occasionally saxicolous). However, as was expected, the predominance of epiphytes is related to the presence of abundant tree species of the deciduous tropical forest. This means that species from different substrate preferences can coexist in the same habitat and vegetation type. For example, on scrubs (“matorral”) we can find epiphytes like *T.juncea*, and *T.ionantha* on cactus or even on rocks; on the other hand, saxicolous species as *T.inopinata* can also grow as epiphytes in the same area.

Most orchids seem to be facultative; for example, species of *Laelia* generally appear as epiphytes, although it has been reported that *L.speciosa* and *L.anceps* can be saxicolous in other regions (Halbinger and Soto-Arenas 1997, Soto-Arenas and Solano-Gómez 2007). Another orchid that can also be facultative is *Mesadenuspolyanthum*; the remaining species here reported are limited to one type of habit (epiphyte, saxicolous, or terrestrial).

Due to the variation in orography, vegetation types and climates found within the RBBM, it is possible that different species can find there an appropriate microclimate/niche to grow. For example, from the species cited in Table 1, orchids like *Laelia* and *Cyrtopodium* species, as well as bromeliads of the genus *Hechtia*, and particularly *Tillandsiaalbida* and *T.inopinata*, are found in xerophytic areas; while in humid environments one may find orchids like *Aulosepalum* and *Sarcoglottis* and bromeliads as *T.violacea*, *T.imperialis*, and *T.deppeana*.

Some species of bromeliads and orchids grow in drier habitats (Gentry and Dodson 1987) which is related to CAM metabolism and economy of water (Benzing 1990, Fontoura and Reiniert 2009). For example, *T.mauryana* and *T.ionantha* grow in xerophytic areas and are adapted to water stress conditions (Benzing 2000). Both have CAM metabolism that may be used for saving water (Benzing 2000, Matiz et al. 2013).

Orchid diversity is considered low in arid areas because there are limiting factors, like water and type of soil that are essential for mycorrhizae formation and seed germination. Even in rocky sites, organic soil matter is usually low or almost nonexistent and hence a limitation (Hágsater et al. 2005). Nevertheless, most of the species reported here are terrestrial and saxicolous. The epiphytic orchids depend not only on the mycorrhizae, but also on the characteristics of trees’ bark and the microclimatic gradients generated by their height (Benzing 1990, Shaw 2004), which could explain the low diversity presented by this habit.

### Species in some risk category

A total of nine endangered or protected species are included in Table 1 (IUCN, CITES, NOM-059-SEMARNAT). The NOM-059-SEMARNAT only proposed *Tillandsiafestucoides* and *Laeliaspeciosa* as species subject to special protection; *T.imperialis* is referred to as a threatened species, and is locally used in beverages (as food or as medicine) by people in Hidalgo (Hornung-Leoni 2011 a,b). *Laeliaanceps* is also mentioned as an endangered species and *L.gouldiana* as an extinct one (*in situ*). On the other hand, the IUCN list only considers *T.ionantha* as a Least Concern (LC) category, and CITES (2017) includes species like *T.mauryana*, *L.anceps*, *L.gouldiana* and *L.speciosa* as Endangered and states that for *Laelia*, the main problem is its use as an ornamental plant subjected to extraction of individuals from the wild for the horticultural trade.

*Tillandsiamauryana* is considered at risk in CITES appendices (2017). According to Espejo-Serna et al. (2004), this species is saxicolous and endemic to Mexico, for the states of Hidalgo, Jalisco, Oaxaca, and Zacatecas. More recently, Espejo-Serna and López-Ferrari (2018) considered *T.mauryana* endemic to Hidalgo, a fact that we have corroborated after reviewing specimens collected in the field and in herbaria. We detected some areas in the RBBM where the species covers a rocky wall, while in other areas only a few individuals are present. In general, we observed scanty populations of *T.mauryana* in the Reserve (see IUCN category assigned in Table 1). No particular use of this species in local trade was reported here, but due to the small number of these populations and to their potential use as ornamental plants, some kind of protection is needed (see Table 1 for IUCN status). Furthermore, it needs to be protected due to the slow population growth rate and the frequent extraction of rocks in the Reserve area (Valverde-Valdés 2013). For those species that are not yet used by local people, the principal risk is habitat destruction.

In Orchidaceae, *Laelia* has categories subject to protection in both CITES and NOM-059-SEMARNAT, since species are exploited commercially and for cultural uses during local festivities. Nevertheless, they are not included in any of the IUCN categories.

*Laeliagouldiana* is the most exploited species and although it is reported as extinct *in situ* (NOM-059-SEMARNAT), it is common to find it in local houses’ backyards inside RBBM, a preponderance that has probably contributed to perpetuate its existence. For the remaining orchid species reported in the RBBM, there are no known ethnobotanical uses. Terrestrial orchids are recognized as such by very few people and are susceptible to vegetation fires during the dry season and to changes in the use of soil.

### Comparing data. Adding new records

For bromeliads, principally tillandsias were included in the management plan (CONANP 2003), but some of them are synonymous or misidentifications. Three out of six species of the orchids included in the management plan are Laelias [*L.autumnalis*, *L.gouldiana* and *L.speciosa* (Kunt) Schltr]. The last two can be found in NOM-059-SEMARNAT, in the categories of extinct in nature and in special protection, respectively (SEMARNAT 2010).

### Biological and ethnobotanical information about species found in the Reserve

Both families are important because they not only provide biological resources to pollinators, visitors and fauna, which fulfill at least a part of their life cycles in these plants (Benzing 2000, Hornung-Leoni et al. 2013), but because they are also used by local people for ornamental, medicinal and ceremonial purposes (Hornung-Leoni 2011a).

Orchids such as Laelias are considered beautiful, abundant and easy to grow and are commonly used in religious celebrations such as the Día de Muertos (Day of the Dead) (Hágsater et al. 2005). Due to the flowering period of *L.anceps* and *L.gouldiana*, it is common to find them in November as an ornamental element in altars, tombs, and churches. For people who live inside the RBBM, *L.gouldiana* is a well-known and widely cultivated species to be used during November festivities and represents an occasional economic income as well (Soto-Arenas and Solano-Gómez 2007). As for *L.speciosa*, we found only one publication in which food and ornamental uses in Hidalgo are included (Pérez Escandón et al. 2003), even though we could personally verify that it is locally sold at Actopan markets in May. Although García-Peña and Peña (1981) report the use of pseudobulbs of *Cyrtopodiummacrobulbon* to prepare cataplasms, it is not known if local people of the RBBM apply the same use to this or other species.

### Suggestions for conservation

Bromeliads and orchids are important in areas that have a conservation focus. It is clear that areas with species under threat like RBBM are important for Mexico if there is, as we think, the intention to create corridors for plants and pollinators.

Summarizing, bromeliads are important components in RBBM, with predominance of *Tillandsia* species, and with a high component of Mexican endemism inside the Reserve. The number of species of orchids has been underestimated and needs to be explored further in the future. Even if the principal habit is epiphytic, both families represent important elements for species diversity in the RBBM, as well as a dominant component in some habitat types. The endemism inside the RBBM highlights the necessity of preserving this variable area and enhancing environmental education and ethnobotanical studies together with local people.

## Conclusions

The Reserve is an important area for flora protection. Even if the RBBM is the most important reserve in the State, it has hitherto not featured in detailed flora inventories and additions to this list are expected in the near future. This study reports 34 species: ten of these are new registers for the Reserve, seven are new records of bromeliads and three are of orchids. Twenty-six bromeliads and eight orchids of the RBBM were recorded and properly documented for the first time. Moreover, the presence of endemic and rare species, as well as of those in risk categories (NOM-059-SEMARNAT, IUCN and CITES), makes it necessary to implement new strategies to maintain the reserve and increase efforts to update its biodiversity data.

Since an important number of endemic taxa (19 species), as well as rare (16 spp.) and occasional species (7) have been found inside the RBBM, the Reserve plays a determinant role in conserving such species. *Ex situ* and *in situ* strategies for such species may integrate a new plan of management focused on the conservation.

## Supplementary materials

Supplementary material 1 Information about species distribution present in Meztitlán Data type: occurrence Explanation note: In this table extra information is included with details principally about species distribution. This dataset is made available under the Open Database License (http://opendatacommons.org/licenses/odbl/1.0/). The Open Database License (ODbL) is a license agreement intended to allow users to freely share, modify, and use this Dataset while maintaining this same freedom for others, provided that the original source and author(s) are credited.Claudia T. Hornung-Leoni, Yesenia J. Chavarria-Olmedo, Ivón M. Ramírez-Morillo
